# Establishment of Degrees in Commerce

**Published:** 1919-11-29

**Authors:** 


### COMMERCIAL EDUCATION.

### Establishment of Degrees in Commerce.

One subject upon which all are agreed, even at this time of universal discussion and change, is the vital need to maintain the commercial supremacy of the British Empire, and though the subject may be a little foreign to the customary trend of medical thought, yet, in view of the indirect importance to the nation, and particularly to its voluntary healing institutions, we need make no excuse for calling attention to the recently issued appeal by the University of London for subscriptions towards establishing organised higher commercial education and distinct degrees in commerce.

### Fresh Sources of Revenue.

Fresh sources of revenue must be sought outside our own borders, and in foreign markets we shall inevitably encounter the keenest competition with the most highly-trained specialists of the United States, France, Japan, and, in a very short time, Germany. It is no exaggeration to say that these have received an intensive training and an education in commerce such as has hitherto been scarcely even dreamt of in our own country. They must be met with their own weapons if any consistent success is to be achieved, and good though the past reputation of Britain may be, it is only knowledge which can give her continued supremacy. In the published appeal it is pointed out, only too truly, that Education, in the person of the Public School University man, has regarded the Church, the law, the Services, and medicine as the only possible careers, and has looked upon commerce as something remote and unworthy of consideration, with possibly slight reservations in favour of banking, insurance, and the selling of motor-cars. Too many of the older business men have been apt to regard higher education as a "pretty toy" for those only who can afford the time. Education for a business life is not necessarily to be obtained only in the office, the warehouse, and the factory; there is the wider outlook, and the country must have men who can comprehend it.

### Two Opposed Parties.

And thus we have had in the past two unjustifiably and unnecessarily opposed parties on the subject. In actual fact, the schoolboy has never had commerce presented to him in its true light and its essential importance, and the business man is so far justified in that education has followed the natural law of supply and demand. Looking at the question from both sides, it becomes obvious that there is needed simply a *rapprochement* between these two, and it is this that the University of London, as representing the capital of the world's commerce, has set itself to bring about.

Already all plans have been laid openly before the most representative gatherings of commercial men; the fullest consideration and criticism has been invited, and every attempt has been made to incorporate in the final proposals the many helpful suggestions thus received. Also they have made it a cardinal principle of their scheme that not only its initiation but the whole of its future working should have the benefit of the closest possible co-operation with the leading business men.

### Progress and Requirements.

It is unnecessary here to recount lists of meetings and names, but suffice it to say that many of the most representative and respected minds in the country are associated with this movement, and that our own subsidiary opinion in the present proposals form a good working basis upon which to ground an important and valuable development in commercial education. The University has decided to grant the degree of Bachelor of Commerce to candidates who pass the necessary examinations after a course of study lasting normally for three years. Those who desire may afterwards proceed to the degree of Master of Commerce, but only after a minimum of two years' satisfactory practical experience in the particular trade or industry taken up. As explained by the University, it will readily be understood that the foundation and maintenance of an entirely new course of studies of the kind must inevitably involve a heavy outlay both for initial expenses and for upkeep. Without going into details, the annual cost of the various proposals outlined is estimated at £21,750, necessitating the provision of £435,000. Adding to this the capital expenses of building, etc., the total sum required is, in round figures, £500,000.

The appeal has been widely circulated among both parties, the commercial world and the graduates of the University, and from its indirect but nevertheless very definite bearing upon the efficiency and upkeep of the medical institutional world, we would call particular attention to what should, in years to come, prove to have been an all-important development.

